# Cardiovascular risk factors and future risk of Alzheimer’s disease

**DOI:** 10.1186/s12916-014-0130-5

**Published:** 2014-11-11

**Authors:** Renée FAG de Bruijn, M Arfan Ikram

**Affiliations:** Department of Epidemiology, Erasmus MC University Medical Center, Wytemaweg 80, Rotterdam, 3015 CN the Netherlands; Department of Neurology, Erasmus MC University Medical Center, ’s-Gravendijkwal 230, Rotterdam, 3015 CE the Netherlands; Department of Radiology, Erasmus MC University Medical Center, ’s-Gravendijkwal 230, Rotterdam, 3015 CE the Netherlands

**Keywords:** Cardiovascular disease, Imaging markers, Risk factors, Dementia, Alzheimer’s disease

## Abstract

Alzheimer’s disease (AD) is the most common neurodegenerative disorder in elderly people, but there are still no curative options. Senile plaques and neurofibrillary tangles are considered hallmarks of AD, but cerebrovascular pathology is also common. In this review, we summarize findings on cardiovascular disease (CVD) and risk factors in the etiology of AD. Firstly, we discuss the association of clinical CVD (such as stroke and heart disease) and AD. Secondly, we summarize the relation between imaging makers of pre-clinical vascular disease and AD. Lastly, we discuss the association of cardiovascular risk factors and AD. We discuss both established cardiovascular risk factors and emerging putative risk factors, which exert their effect partly via CVD.

## Introduction

Alzheimer’s disease (AD) is the most common subtype of dementia, and has a large patient and societal burden. AD has a complex and multifactorial etiology that involves senile plaques and neurofibrillary tangles [1]. Increasingly, the role of cardiovascular disease (CVD) is also being recognized as an important etiologic hallmark of AD. Indeed, many studies have shown the importance of vascular pathology in AD [2]-[7]. As CVDs have established therapeutic options and the risk factors of CVD are modifiable, focusing on the association between vascular pathology and AD might provide pathways to prevent or delay AD in elderly individuals [8],[9]. In this narrative review, we provide an overview of the current knowledge on the relation between AD and clinical CVDs, imaging markers of pre-clinical CVD, and established and emerging cardiovascular risk factors (Table 1).

**Table 1:** **List of potential vascular factors implicated in Alzheimer’s disease**

Cardiovascular disease	Pre-clinical markers of cardiovascular disease	Established cardiovascular risk factors	Emerging risk factors
Stroke	Intima media thickness	Blood pressure, hypertension, and arterial stiffness	Inflammation
Atrial fibrillation	Carotid plaques	Glucose metabolism and diabetes mellitus	Chronic kidney disease
Coronary heart disease	Atherosclerotic calcification	Hypercholesterolemia	Thyroid function
Heart failure	Lacunae and white matter lesions	Smoking	
	Cerebral microbleeds	Obesity	
	Cerebral microinfarcts	Non-adherence to the Mediterranean diet and low levels of physical activity	
	Retinal vascular changes	Homocysteine	
	Microstructural integrity and connectivity		

## Review

### Cardiovascular disease

CVDs, such as stroke, atrial fibrillation, coronary heart disease (CHD), and heart failure are very common in elderly individuals and have regularly been linked to AD. This association might be due to shared risk factors between CVDs and AD, but there might also be a direct causal association as cardiac disease causes hypoperfusion and microemboli, which have been implicated in the etiology of AD [10],[11]. In the following sections, we discuss current evidence relating common CVDs with risk of AD.

#### Stroke

Clinical stroke has often been associated with an increased risk of subsequent dementia, but this is by definition then termed `post-stroke dementia’ or `vascular dementia’ [12]. Such terminology hampers thorough investigation of the role of clinical stroke in AD. Therefore, important evidence implicating stroke in the etiology of AD comes from studies investigating asymptomatic or `silent’ stroke, which are often lacunae. Numerous studies have shown that lacunae strongly increase the risk of dementia, including AD [13]-[15]. Moreover, white matter lesions, which also represent ischemic brain damage, are also associated with cognitive impairment and AD [16],[17]. These findings suggest that stroke is causally involved in the etiology of dementia. Mechanisms underlying this association include the following. Firstly, stroke causes loss of neuronal tissue, which might enhance the degenerative effect of neuronal tissue loss as a result of amyloid and tau pathology [15]. Secondly, it has been suggested that cerebrovascular disease directly influences amyloid pathology as a result of accelerating amyloid β production or hampering amyloid β clearance [3],[18], although studies on these pathways remain inconsistent [3],[18]-[21].

#### Atrial fibrillation

Several studies have shown that individuals with atrial fibrillation (AF) more often have AD and are at an increased risk of AD [22]-[24]. Because AF causes embolisms that could lead to stroke, the relation between AF and AD might be explained by clinical or silent stroke [10],[25]-[27]. Accordingly, a meta-analysis showed that a consistent relation between AF and a higher risk of dementia was restricted to individuals with stroke [23]. However, another study found that stroke-free individuals with AF performed worse on memory and learning tasks, and had a reduced hippocampal volume [28]. Both memory function and hippocampal volume are strongly related to AD, which suggests there might be additional pathways explaining the association between AF and AD [29]. One hypothesis is that cerebral hypoperfusion in AF causes damage to nerve cells, and thereby contributes to the etiology of AD [23],[25]-[27]. Another hypothesis is that AF directly influences AD neuropathology, such as senile plaques and neurofibrillary tangles, but evidence for this explanation remains scarce [30].

#### Coronary heart disease

CHD is the most common type of heart disease, and one of the major causes of death worldwide [31]. CHD includes angina pectoris, myocardial infarction (MI), and coronary revascularization procedures. The relation between CHD and AD remains difficult to disentangle because of strong competing risks of death; several studies showed that CHD is related to cognitive impairment or AD [32],[33], whereas others found no association [34],[35]. The Rotterdam Study showed that unrecognized MI was associated with the risk of AD, whereas recognized MI was not [36]. Explanations linking CHD with AD include shared etiology, as atherosclerosis plays an important role in both CHD and AD [26],[27]. This hypothesis is corroborated by findings from the Cardiovascular Health Study, which showed that peripheral artery disease, another manifestation of atherosclerosis, was also strongly associated with an increased risk of AD [32]. Furthermore, CHD might relate to AD through diminished cardiac function, hypoperfusion, and emboli [10],[25]-[27].

#### Heart failure

Heart failure represents a condition in which the pumping function of the heart is diminished and unable to supply the body with sufficient blood flow. Heart failure has been associated with cognitive impairment and AD [37]-[39]. A Swedish study found that heart failure was related to an increased risk of dementia, including AD [37]. The same study also found that treatment with anti-hypertensive drugs slightly reduced this risk. The Framingham Offspring Study showed that even in individuals without clinical heart failure, lower cardiac function was related to lower brain volume, an important hallmark for dementia [40]. The pathways explaining the role of heart failure in the etiology of AD are similar to those of AF; heart failure results in hypoperfusion of the brain, which leads to hypoxia and damage to nerve cells [3],[4],[25]-[27]. Additionally, heart failure increases the risk of emboli and microvascular pathology, such as white matter lesions and lacunae, which in turn are related to an increased risk of dementia [10],[25]-[27].

### Pre-clinical markers of cardiovascular disease

Cardiovascular pathology gradually accumulates over years before manifesting as a clinical event. Similarly, AD pathology also accumulates over decades before clinical symptoms occur. Consequently, several studies have sought to investigate how such pre-clinical pathology relates to cognitive decline and AD.

#### Pre-clinical markers of large vessel disease

Using various imaging techniques, it is possible to assess markers of pre-clinical large vessel disease. Intima media thickness (IMT) and carotid plaque are measures of atherosclerosis in the carotid artery, which can be obtained via ultrasonography. Both IMT and carotid plaque are more prevalent in patients with dementia and AD than in cognitively healthy individuals [41]. Moreover, both measures are related to increased cognitive decline in patients with AD [42]. Additionally, several population-based studies have shown that individuals with the highest IMT measures have an increased risk of incident dementia, including AD [32],[43],[44]. Carotid plaque scores were also associated with an increased risk of AD in one study, but this association lacked statistical significance [44]. Another marker of pre-clinical large vessel disease is calcification volume in the atherosclerotic plaque, which can be assessed using computed tomography (CT). Although calcification is only part of the plaque, it is a suitable measure of the underlying plaque burden [45]. CT has the disadvantage of radiation exposure, but CT measures of atherosclerotic calcification are more observer-independent than ultrasonography measures. Few studies have investigated the relation between CT-derived atherosclerotic calcification and dementia, but some studies found that larger calcification volumes in the coronary arteries, aortic arch, and carotid arteries relate to worse cognitive performance [46],[47]. Moreover, larger calcification volume was associated with smaller brain tissue volumes and worse microstructural integrity of the white matter, which are both factors related to an increased risk of AD [46]. Mechanisms linking carotid large vessel disease to AD include sub-clinical cerebral small vessel disease (see below), hypoperfusion, or shared etiology [3],[4],[6].

#### Pre-clinical markers of cerebral small vessel disease

Abundant evidence shows that structural imaging markers of cerebral small vessel disease, such as lacunae and white matter lesions, are related to cognitive impairment or AD [15]-[17],[48]-[50]. Additionally, brain atrophy, which is an established marker of dementia and AD, is partly influenced by CVD [48],[51],[52]. Cerebral microbleeds (CMBs) are an emerging vascular marker with great promise for AD research. Both amyloid β and vascular pathology are related to the etiology of CMBs, and therefore a link between CMBs and incident AD seems plausible [53]-[55]. However, this association still needs to be confirmed in longitudinal studies. In recent years, it has also become possible to visualize cerebral microinfarcts using high-field magnetic resonance imaging (MRI) scanners, such as 7 T scanners. The role of these microinfarcts in AD remains unclear, but is expected to be the focus of research in coming years [56],[57]. Although it is possible to measure markers of cerebral small vessel disease, direct visualization of the small cerebral arterioles *in vivo* remains difficult. Retinal imaging provides an easy tool to visualize retinal vessels that originate embryologically from the same tissues as cerebral vessels. Thus retinal imaging provides a possibility to study the small vessels of the brain *in vivo*. Retinal vessel diameter has been associated with white matter lesions, infarcts, brain atrophy, and an increased risk of vascular dementia [58]-[60]. Although a recent case-control study also found a link between AD and retinal microvascular changes [61], there is currently no evidence relating retinal vessels to an increased risk of AD longitudinally.

#### Measures of brain connectivity

In recent years, development of newer imaging techniques has allowed quantification of more subtle brain pathology such as changes in brain connectivity. Diffusion tensor imaging (DTI) assesses the microstructural integrity of the white matter, and studies have suggested that DTI markers reflect a very early stage of vascular brain pathology. Consequently, several studies have shown loss of microstructural integrity in early AD or even in mild cognitive impairment (MCI) [62]-[64]. However, longitudinal studies relating DTI markers to incident AD are still largely lacking. Another novel MRI technique is resting-state functional MRI, which measures brain function by functional connectivity at rest. Several studies have shown that functional connectivity is altered in patients with MCI and AD [65]-[69], but again, robust longitudinal data are still lacking. Moreover, the role of cardiovascular risk factors in functional MRI remains unclear.

### Cardiovascular risk factors

In addition to clinical CVDs (see above), risk factors of CVD have also been implicated in AD. The causal pathway of these risk factors might be associated with clinical disease, but there is also evidence directly linking cardiovascular risk factors with AD.

#### Blood pressure, hypertension, and arterial stiffness

Several studies have related hypertension to brain atrophy, white matter lesions, and neurofibrillary tangles [70]-[72]. Therefore, an association between hypertension and AD is conceivable. Nonetheless, this association is complex and differs with age [73]. Several studies show mid-life hypertension to be related to an increased risk of AD [74]-[77], whereas other studies failed to find an association between late-life hypertension and dementia. In fact, some studies even suggest low blood pressure might be related to AD [73]. These inconsistencies have yet not been elucidated, but it is suggested that blood pressure decreases in the years before clinical onset of dementia because of reduced physical activity and lowered body weight. Further research is still necessary to verify this hypothesis [27].

A measure closely related to blood pressure and hypertension is arterial stiffness, which can be measured as increased pulse pressure or elevated pulse wave velocity. The difficulty in investigating arterial stiffness lies in the fact that it can be caused by hypertension as well as leading to hypertension [78],[79]. Arterial stiffness results in increased pulsatile pressure, causing damage to the microvascular system of the brain [80], which in turn causes cognitive decline [80]. Indeed, some studies found a relation between higher pulse pressure or higher pulse wave velocity and an increased prevalence and risk of cognitive decline or AD [81]-[83]; however, others could not demonstrate such an association [84],[85].

#### Glucose metabolism and diabetes mellitus

Type 2 diabetes mellitus (T2DM) is a complex disorder, in which insulin resistance leads to higher circulating blood glucose levels, which in turn lead to microvascular damage in various organs. In the brain, T2DM has been associated with infarcts and atrophy [86],[87]. Accordingly, many studies have confirmed that the risk of dementia and AD is higher in individuals with T2DM [88]. Furthermore, the risk of AD is also increased in individuals with borderline T2DM, that is, pre-diabetes [89]. Besides microvascular damage, other potential mechanisms relating T2DM with AD are direct neurotoxicity due to increased glucose and insulin levels. A higher circulating blood glucose level is toxic to nerve cells, as it causes protein glycation and oxidative stress [88]. Insulin is involved in amyloid β clearance from the brain, and higher levels of insulin could disrupt this metabolism, leading to increased amyloid β burden [88].

#### Hypercholesterolemia

Given the role of cholesterol in the clearance of amyloid β, hypercholesterolemia has been suggested as a risk factor for AD. Support for this hypothesis comes from a recent imaging study showing higher cholesterol levels to be related to higher amyloid β levels [90]. Similarly, *apolipoprotein E* ε4-carrier status, one of the most important genetic risk factors of AD, is related to increased cholesterol levels [91]. However, results of epidemiological studies on the association between hypercholesterolemia and AD have been inconsistent. Some studies found that hypercholesterolemia in mid-life was associated with an increased risk of AD, whereas in late life there was no association [92]. An explanation is that a high cholesterol level in mid-life is a risk factor of AD, whereas lower cholesterol levels in late life probably reflect pre-clinical disease, as lifestyle and dietary habits change in individuals with sub-clinical dementia.

#### Smoking

Various longitudinal studies have established smoking as a risk factor for dementia and AD [93]. Both the Rotterdam Study and the Honolulu-Asia Aging Study found that the risk of dementia in smokers was higher than that in non-smokers [94],[95]. Furthermore, the Honolulu-Asia Aging Study found that number of pack-years was related to amyloid burden in the brain in a dose-response manner [95]. Smoking contributes to atherosclerosis, and has been related to cerebral small vessel disease [49],[96]. Additionally, tobacco contains many neurotoxins, which might cause direct neuronal damage [97]. However, the exact mechanisms underlying the relation between smoking and dementia require further investigation.

#### Obesity

Similar to hypertension and increased cholesterol levels, the association between obesity and risk of dementia and AD changes with age [98]-[100]. Obesity in mid-life is associated with an increased risk of dementia and AD, whereas in older age a higher body weight seems to have a protective effect [100],[101]. Individuals with sub-clinical dementia gradually lose body weight due to altered lifestyle and lowered food intake, and thus low body weight might also be an early symptom of dementia [98]-[100]. In contrast, mid-life obesity increases the risk of many chronic diseases, including vascular diseases, and could be related to an increased risk of dementia and AD via those pathways [101].

#### Mediterranean diet and physical activity

The Mediterranean diet is characterized by a high intake of vegetables, fruits, cereals, and unsaturated fatty acids, a moderate intake of fish, poultry, eggs, red wine, and dairy products, and a low intake of saturated fats and red, processed meats [102]. Adherence to a Mediterranean diet has shown to reduce vascular disease and vascular risk factors, and to lower inflammation and oxidative stress [103]. Two recent meta-analyses concluded that adherence to a Mediterranean diet might reduce the risk of AD [104],[105]. However, the number of studies with long follow-up is limited, and further research is necessary to confirm the potential protective effect of the Mediterranean diet on AD.

Besides dietary habits, another potential modifiable factor to reduce AD risk is physical activity [9],[106]. Physical activity is inversely associated with CVD and diabetes, and could therefore also reduce the risk of AD [107],[108]. Alternatively, physical activity could have a direct protective effect on the risk of dementia, as it improves cerebral perfusion and increases neurogenesis [109],[110]. Several epidemiological studies have associated a higher level of physical activity with a reduced risk of dementia or cognitive decline [111]-[115]. However, most of these studies had relatively short follow-up, and studies with long follow-up periods have yielded inconsistent results [115],[116]. For both physical activity levels and the Mediterranean diet, the possibility of reverse causality explaining short-term associations needs to be considered [117].

#### Homocysteine

Plasma homocysteine levels reflect folate and vitamin B12 status, and are related to renal function. Increased homocysteine levels are associated with vascular disease, and might have an effect on amyloid β and tau phosphorylation. Consequently, high plasma homocysteine levels have been related to an increased risk of AD [118]. Imaging and autopsy studies showed that increased homocysteine levels were associated with brain atrophy and neurofibrillary tangles [119],[120]. However, not all studies concur with these results. A recent study found that plasma homocysteine levels were not related to AD, after adjusting for folate or vitamin B12 deficiency and renal dysfunction [121]. Further studies are needed to unravel this association.

### Emerging risk factors

In addition to the classic vascular risk factors, there are other emerging risk factors that have been implicated in AD, partly by vascular mechanisms.

#### Inflammation

Various inflammatory markers have been related to an increased risk of dementia, including AD [122]-[124]. Astrocytes and microglia activate the neuronal immune system in response to pathogens such as infection and vascular pathology [125],[126]. Several studies showed that senile plaques in the brains of patients with AD and of AD transgenic mice models were surrounded by an increased number of activated microglia [127]. Amyloid β also activates the neuronal immune system, and might cause a chronic inflammatory reaction that has a toxic effect on nerve cells [126]. Moreover, recent genetic studies have uncovered various genes for inflammation and immune response that seem to be associated with AD [128]. However, there have been no major population-based cohort studies studying inflammation in AD, and trials studying the effect of immunotherapy on AD have not yet been successful [126]. Hence, further studies are required to elucidate the exact role of inflammation in AD.

#### Chronic kidney disease

In recent years, various studies have focused on the association between chronic kidney disease (CKD) and cognitive decline or AD. Most [129]-[133], but not all [134] of these studies found that low kidney function was related to an increased risk of dementia, AD, or cognitive decline. These inconsistencies might be due to methodological discrepancies: different measures of kidney function were used, and there was a large variation across the study populations examined [132]. Mechanisms linking CKD and dementia include shared risk factors (such as hypertension, arterial stiffness, smoking, and obesity) and direct consequences of CKD (such as chronic inflammation, hemodynamic changes, anemia, and uremic toxins) [129]. However, these pathways are not well established, and should be investigated further.

#### Thyroid function

Thyroid hormone is important for brain function, and thyroid dysfunction is a potentially reversible cause of cognitive impairment [135]. Thyroid hormone is involved in amyloid precursor protein (APP) regulation. Animal studies have shown that APP expression is increased in hypothyroidism, which leads to higher amyloid β levels [135]. In addition, thyroid dysfunction is associated with CVD, and could therefore influence AD pathology indirectly [135]. Lastly, thyroid hormone levels alter as a consequence of AD pathology through reduction in thyrotropin releasing hormone secretion [136]. Observational studies have shown both hypothyroidism and hyperthyroidism to be related to AD, but not all studies could establish an association [136]-[140].

## Conclusion

In conclusion, there is abundant and converging evidence showing that CVDs and cardiovascular risk factors play an important role in the etiology of AD. While for some of these factors the mechanisms linking to AD are clear, for others the association with AD is more complex and needs further research to be completely unraveled. Nevertheless, given that these vascular factors are currently the only known modifiable risk factors for AD, the possibility of intervening with these factors to prevent or delay AD merits more dedicated research.

## Authors’ contributions

RB and MAI both made substantial contributions to conception and design of the manuscript, and were involved in drafting the manuscript and revising it critically for important intellectual content. Both authors read and approved the final manuscript.

## Abbreviations

AD: Alzheimer’s disease
AF: Atrial fibrillation
APP: Amyloid precursor protein
CHD: Coronary heart disease
CKD: Chronic kidney disease
CMBs: Cerebral microbleeds
CT: Computed tomography
CVD: cardiovascular disease
DTI: Diffusion tensor imaging
IMT: Intima media thickness
MCI: Mild cognitive impairment
MI: Myocardial infarction
MRI: Magnetic resonance imaging
T2DM: Type 2 diabetes mellitus
